# A Case of Rheumatic Carditis, Suddenly Fatal

**Published:** 1873-08

**Authors:** H. A. Johnson

**Affiliations:** M. D. and Prof., Chicago, Ill.; No. 4 Sixteenth St.


ARTICLE V.—*A Case of Rheumatic Carditis, Suddenly Fatal.* By H. A. JOHNSON, M.D. and Prof., Chicago, Ill.*

I saw Mrs. S., æt. 31, first on January 9th, 1873, early in the afternoon. I found her suffering from ill-defined muscular soreness, especially over the abdomen and in the back—bowels not disturbed—skin moist—pulse slightly accelerated—tongue moist and white. I prescribed the following:

R. Potass. Bromid, - - - - - dr. iij.
Aq. Camph., - - - - - f oz. iv.
M. ft. sol. Sig. One tablespoonful every three hours.

Jan. 10. Saw her in the afternoon. She passed a restless night. Some nausea this morning, which she attributes to the medicine; supposed that she had taken morphine or opium. Pain more decidedly rheumatic — hips and knees sore — motion difficult. Menses came on during the night or this morning, several days earlier than expected. Begs for something to allay pain, which was now almost unbearable. I directed the following:

R. Hydr. Sub Mur., - - - - - gr. jv (4)
Morph. Sulph., - - - - - gr. j.
Sodæ Bi Carb., - - - - - scr. j.
M. Ph. pulv. No. vj. (6).
S. One every three hours.

the experiment was made the patient heard a tone of 100,000 v. s. distinctly, after the operation, the extreme limit before the operation having been only a tone of 35,000 v. s.

* Received too late for insertion in its proper place.

Jan. 11, P. M. Pain still continues in hips and knees. She attributes a part of it to the advent of the menses. Condition of the stomach improved. Has rested and slept some under the influence of the powders of morphine, sub muriate and soda, but has not taken them as often as directed. I ordered them to be continued as often as necessary to relieve pain.

Jan. 12. Bowels have moved—probably from the effect of the powders, though not all of the four (4) grs. of calomel has yet been taken. Pains more general—complains of shoulders. Skin moist—tongue coated, white, moist—urine sufficient in quantity, and normal in quality—pulse 96, or a little below 100, per minute. Made no change in the treatment.

Jan. 13. The condition about the same. Rheumatic difficulty more decidedly migratory. The powders of calomel, morphia and soda, prescribed on the 10th, have been renewed—without my direction, however. Does not take much food. Has all the time been worse during the night than during the day. No change in treatment.

Jan. 14. I had been quite sick for two or three days, and on the 14th was unable to leave my bed. I heard nothing from the case till the morning of the 15th, when I learned from Dr. F. H. Davis and Dr. Lester Curtis that they had both seen her on the evening of the 14th. Dr. Curtis made the following prescription:

R. Potass. Bromid, - - - - - - dr. ij. ss.

Aqua, - - - - - - } *aa* f oz. j.

Syr. Cort. Aurant., - - - }

M. S. One teaspoonful every hour as directed.

I am told that she took one dose of the mixture prescribed by Dr. Curtis, after which Dr. Davis was sent for. He ordered the following:

R. Potass. Bromid, - - - - - dr. iv.
Cryst. Carbolic Acid, - - - - gr. vj.
Glycerine, - - - - - - f oz. ss.
Aqua, - - - - - - f oz. iij. ss.

M. One teaspoonful every three hours.

This was continued during the night, and till I saw her on the 15th. The powders were continued as they were judged necessary to allay pain.

I saw her about 2 P. M. They said that Dr. Davis' medicine had given her great relief. Some muscular tenderness yet over the chest and in the shoulders—pulse about 100, regular, and moderately full. Directed the continuance of the bromide of potass. and carbolic acid solution. Saw her again in the evening.

Jan. 16. Less positive pain, but had a restless night—only "snatches of sleep," as she expresses it. Has not taken the powders since yesterday. Directed the following:

R. Tr. Opii Camph., - - - - - f oz. j.

S. Half teaspoonful every two hours, if awake.

Continue the bromide and carbolic acid solution. She only took one dose of the paregoric.

In the evening she begged for something to relieve the pain, which had become much more decided, especially in the limbs and joints—could hardly move in bed. I gave her a hypodermic injection of morph. $\frac{1}{4}$ gr. and atropin $\frac{1}{60}$ gr. She went to sleep in about five minutes, and slept quietly till three or four o'clock in the morning, after which she was restless—slept between seven and eight hours.

Jan. 17. Found her in the morning absolutely helpless—arms, hands, fingers, hips, knees, ankles and toes even, tender, and most of the joints somewhat swollen—skin hot, but still moist—tongue moist—urine scanty—pulse over 100, hard, small but regular—heart sounds normal in quality. Directed the following:

| | | |
|---|---|---|
| R. | Tr. Colchici, - - - - - | f dr. j. |
| | Potass. Bi Carb., - - - - | dr. ij. |
| | Potass. Cit., - - - - - | dr. iij. |
| | Aq. Camph., - - - - - | f oz. ij. |
| | Syr. Limon., - - - - - | f oz. iv. |

M. ft. sol. Take one tablespoonful every two hours.

This prescription was continued during the day. She was a little flighty when left alone, but recovered perfectly when spoken to, and answered intelligently questions addressed to her. In the evening, about the same. Has not slept during the day—in fact, not much since 3 or 4 A. M. Gave again a hypodermic injection of morph. $\frac{1}{5}$ gr. and atropin $\frac{1}{75}$ gr.

January 18, morning. Limbs less painful and tender, can move them a little—pulse about 95—skin moist, and cooler than last night—slept between six and seven hours under the influence of the injection of morphine and atropine. I directed the colchicum mixture to be continued.

P. M. Bowels have moved freely during the day, copious watery discharges—limbs better, can use them quite well—complains of pain most in right shoulder—is very weary and wants sleep. Discontinued colchicum mixture, and gave, between 3 and 4 P. M., a hypodermic injection of morph. $\frac{1}{3}$ gr. and atropin $\frac{1}{75}$ gr.

January 19, morning. Found her free from pain in joints—had slept several hours during the night, but was still restless, anxious—talking a little incoherently, but answering intelligently when spoken to—pulse about 100—urine scanty—micturition difficult and painful. Directed the following:

| | | |
|---|---|---|
| R. | Potass. Acet., - - - - - | dr. iij. |
| | Potass. Bromid, - - - - - | dr. ij. |
| | Spts. Nit. Dulc., - - - - - | f oz. j. |
| | Syr. Limon., - - - - - - | f oz. v. |

M. ft. sol.

Take one tablespoonful every two hours till relieved.

P. M. Has passed urine freely and without pain—skin quite moist, but hot—pulse more frequent, 115 per minute, regular. Suspected cardiac trouble—found heart sounds normal in quality—no articular pain except in right shoulder, and this not severe. Discontinued diuretic, and ordered the following:

| | | |
|---|---|---|
| R. | Sat. Tr. Rad. Aconite, - - - - | f dr. j. |
| | Morph. Sulph., - - - - - | gr. ij. |
| | Syr. Simp., - - - - - | f oz. ij. |

M. ft. sol.

Take one teaspoonful every two hours.

Saw her three times during this day. She slept during the afternoon and evening only a few minutes at a time, and then would start up suddenly, saying something irrelevant, but upon being spoken to, becoming perfectly conscious.

January 20, morning. Pulse 90, soft, moderately full, regular in rhythm. Mr. S. says that after my visit last night the pulse went up to 120 per minute. Skin moist, not so hot as during the day of the 19th—urine free in quantity—tongue moist—little or no pain anywhere—stomach tolerates milk.

6 P. M. Pulse 84, regular, soft, moderately full—skin moist and cool, but not cold, apparently normal in its qualities—has had no pain during the day—during the last twenty hours has had no continuous quiet sleep. Has been taking during the day half teaspoonful doses of the aconite and morphine mixture. For the purpose of procuring sleep, I directed the following:

| | | |
|---|---|---|
| R. | Chloral Hydrate, - - - - | dr. j. |
| | Syr. Simp., - - - - - - | f oz. ij. |
| | Glycerine, - - - - - | f oz. j. |
| | Chloroform, - - - - - - | f dr. ss. |

M. ft. sol.

S. Dose, one tablespoonful.

Of this mixture, one tablespoonful was ordered to be given every hour till she slept. She took but one dose, and soon afterwards became wildly delirious.

At 10 P. M., or about that time, I saw her again. The pulse was then 104 and somewhat irregular, small, weak—breathing oppressive, panting—vomited fluid matters, apparently milk and mucus, immediately after I came in—bowels also moved, did not see the stool—she was helped out of bed—did not seem to have any pain. In answer to questions, complained only of nausea and oppression at epigastrium. She had been now twenty-four hours without continuous sleep, and I gave, for the purpose of producing it, a hypodermic injection of morphine, gr. $\frac{1}{5}$, and atropin, gr. $\frac{1}{75}$. She went to sleep in about eight minutes—the pulse became more regular at 102 per minute—breathing easier, but not free and full—the temperature of the skin seemed to be normal, and was neither dry nor excessively moist.

At 3 A. M. of the 21st, I was sent for, and upon arriving I found her dead. Mr. S. said that she had slept continuously from the time I left—that between 2 and 3 A. M. they turned her in bed, and that she suddenly ceased to breathe. She had nourishment as the stomach would tolerate it. During the last day or two milk was grateful, and she took as much of it as she liked.

Throughout her sickness, she was worse at night.

NO. 4 SIXTEENTH ST., *Feb.* 1, 1873.

